# RE-AIM implementation outcomes and service outcomes: what’s the connection? results of a cross-sectional survey

**DOI:** 10.1186/s12913-023-10422-w

**Published:** 2023-12-15

**Authors:** Christina R. Studts, Bryan Ford, Russell E. Glasgow

**Affiliations:** 1 Department of Pediatrics, Adult and Child Center for Outcomes Research and Delivery Science (ACCORDS), University of Colorado Anschutz Medical Campus, 1890 N. Revere Ct., Aurora, CO 80045 USA; 2 Department of Family Medicine, Adult and Child Center for Outcomes Research and Delivery Science (ACCORDS), University of Colorado Anschutz Medical Campus, Aurora, CO USA

**Keywords:** Implementation, Implementation outcome, Quality of care, RE-AIM, Implementation outcomes framework, Health services research, On-line survey

## Abstract

**Background:**

Implementation science and health services outcomes research each focus on many constructs that are likely interrelated. Both fields would be informed by increased understanding of these relationships. However, there has been little to no investigation of the relationships between implementation outcomes and service outcomes, despite general acknowledgement that both types of outcomes are important in the pathway to individual and population health outcomes. Given the lack of objective data about the links between implementation and service outcomes, an initial step in elucidating these relationships is to assess perceptions of these relationships among researchers and practitioners in relevant fields. The purpose of this paper is to assess perceived relationships between Reach, Effectiveness, Adoption, Implementation, and Maintenance (RE-AIM) framework outcomes and service outcomes, testing five *a priori* hypotheses about which perceived relationships may be strongest.

**Methods:**

A cross-sectional online survey was administered to a convenience sample of implementation scientists, health services researchers, and public health and medical practitioners from a variety of settings. Respondents provided information on their discipline, training, practice and research settings, and levels of experience in health service outcomes research, implementation science, and the RE-AIM framework. Next, they rated perceived relationships between RE-AIM and service outcomes. Repeated measures analysis of variance were used to test *a priori* hypotheses. Exploratory analyses assessed potential differences in mean ratings across groups of respondents categorized by discipline, setting, and levels of implementation science, health services, and RE-AIM experience.

**Results:**

Surveys were completed by 259 respondents, most of whom were employed in academic and medical settings. The majority were doctoral-level researchers and educators or physicians. Reported levels of experience with implementation research, health services research, and the RE-AIM framework varied. The strongest perceived relationships overall were between Implementation/Fidelity and Effectiveness (as a service outcome); Maintenance and Efficiency; Reach and Equity; Adoption and Equity; Implementation/Adaptation and Patient-Centeredness; Adoption and Patient-Centeredness; and Implementation/Fidelity and Safety. All but one of the a priori hypotheses were supported. No significant differences in ratings of perceived relationships were observed among subgroups of respondents.

**Conclusions:**

This study is an initial step in developing conceptual understanding of the links between implementation outcomes, health services outcomes, and health outcomes. Our findings on perceived relationships between RE-AIM and services outcomes suggest some areas of focus and identify several areas for future research to advance both implementation science and health services research toward common goals of improving health outcomes.

**Supplementary Information:**

The online version contains supplementary material available at 10.1186/s12913-023-10422-w.

## Background

**Fig. 1:**
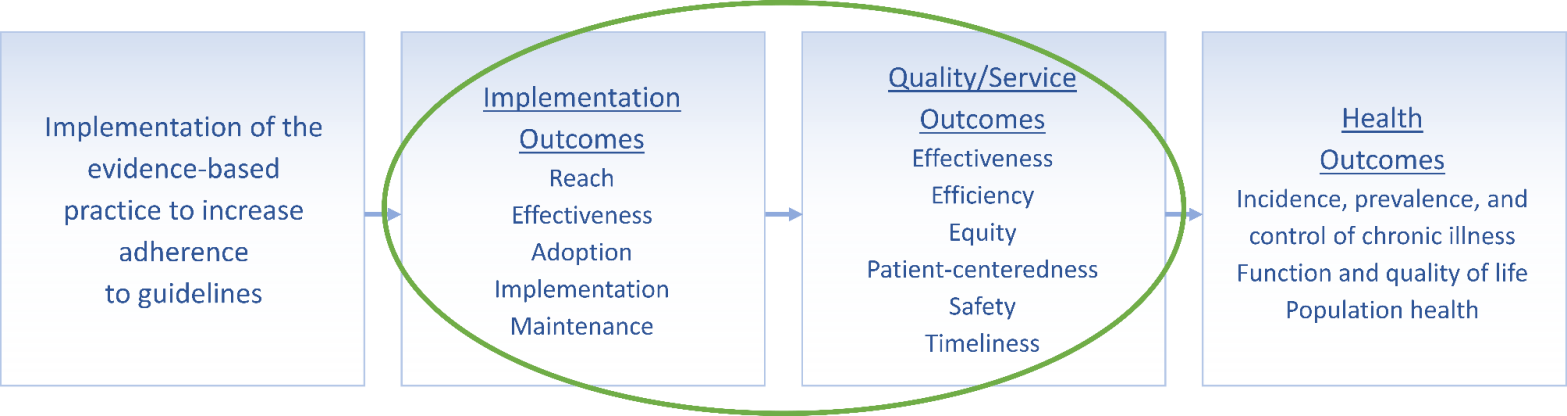
Posited general relationships between RE-AIM implementation outcomes, service outcomes, and health outcomes. *Note*: Figure adapted from the original Implementation Outcomes Framework and used with permission from Enola Proctor [6]

Science advances through investigation and understanding of relationships among different constructs and variables. In implementation science, there has been general consensus that the use of evidence-based interventions [1], supported by specific implementation strategies [2], is expected to lead to short-term and more distal implementation outcomes [3, 4] and individual and population health outcomes (see, for example, the Implementation Research Logic Model) [5]. This conceptual understanding was reinforced in Proctor’s Implementation Outcomes Framework (IOF [6]; Fig. 1), proposing three sequential and related sets of outcomes: implementation outcomes (e.g., adoption, penetration); service outcomes (e.g., equity, efficiency, safety); and more distal client and population health outcomes (e.g., satisfaction, function, symptomatology). According to the IOF, use of effective implementation strategies to deliver effective interventions leads to one or more implementation outcomes, which in turn lead to one or more service outcomes, which finally lead to one or more individual or population health outcomes.

A considerable amount of research has investigated relationships among implementation strategies [7], mechanisms [8, 9], and implementation outcomes [10], but the links among implementation outcomes, service outcomes, and client outcomes have been underexplored. In particular, the outcomes labeled by Proctor et al. [6] as “service outcomes”—including equity, safety, effectiveness, patient-centeredness, efficiency and timeliness—are rarely discussed in the implementation science literature. These outcomes were drawn from a 2001 report from the Institute of Medicine (IOM; now the National Academies of Sciences, Engineering, and Medicine) identifying them as key characteristics of quality health care [11, 12]. They are widely viewed as health system goals, characterized by the Agency for Healthcare Research and Quality as the “Six Domains of Health Care Quality” [13] and utilized by the Institute for Healthcare Improvement and other healthcare organizations [14]. In this paper, we rely on the definitions provided by the IOM and refer to them as service outcomes (see Table 1).

**Table 1:** Definitions of RE-AIM outcomes and service outcomes used in the survey

Outcomes	Definition
**RE-AIM** ^a^	
Reach	The proportion and representativeness of individuals in the target population who receive the evidence-based program. [WHO is intended to benefit and who actually participates or is exposed to the intervention?]
Adoption	The proportion and representativeness of settings and staff that adopt the evidence-based program. [WHICH settings and staff are intended to use the program, and which actually use it?]
Implementation/Fidelity	How consistently the evidence-based program is delivered as intended.
Implementation/Adaptation	Whether changes or modifications are made to the evidence-based program.
Implementation/Cost	How much it costs to deliver the program, including expenses for personnel, materials, training, and supervision.
Maintenance	The proportion and representativeness of settings and staff that that continue to deliver the evidence-based program over time. [WHICH settings and staff continue the program over time?]
**Service** ^**b**^	
Effectiveness ^a^	Providing care that is based on systematically acquired evidence demonstrating that a program or intervention produces better outcomes than alternatives—including the alternative of doing nothing.
Efficiency	Avoiding waste, including waste of equipment, supplies, ideas, and energy.
Equity	Providing care that does not vary in quality because of personal characteristics such as gender, ethnicity, geographic location, and socioeconomic status.
Patient-Centeredness	Providing care that is respectful of and responsive to individual patient preferences, needs, and values and ensuring that patient values guide all clinical decisions.
Safety	Avoiding injuries to patients from the care that is intended to help them.
Timeliness	Reducing waits and sometimes harmful delays for both those who receive and those who give care.

^a^Effectiveness is a RE-AIM outcome and a service outcome. The survey included Effectiveness as both. In analyses, Effectiveness is used only as a service outcome to decrease redundancy

^b^Service outcome definitions are drawn from the Institute of Medicine (2001) report: *Crossing the Quality Chasm: A New Health System for the 21st Century*

Recently, the implementation outcomes defined in the IOF were “mapped on” to the Reach, Effectiveness, Adoption, Implementation, and Maintenance (RE-AIM) framework outcomes [15], offering implementation researchers an integrated conceptualization that incorporates unique strengths of both frameworks (e.g., attention to reach and adaptation from RE-AIM; inclusion of acceptability, appropriateness, and feasibility in the IOF). Our research team is actively engaged in applying the RE-AIM and PRISM framework (“expanded RE-AIM”) [8, 16, 17] and supporting its use beyond our own work. We are especially interested in how the different RE-AIM implementation outcomes (Table 1) relate to various service outcomes.

To our knowledge, there is no review, body of literature, or even single rigorous study that has systematically assessed RE-AIM outcomes in relationship to the six IOM service outcomes [15, 18]. Given the lack of objective data about relationships among these outcomes, we opted to assess the perceptions of different types of researchers and practitioners about these links as an initial step in advancing our conceptual understanding of these relationships. We created an on-line survey and recruited a diverse sample of respondents, including researchers and practitioners in implementation science and health service outcomes research. We aimed to recruit respondents from both healthcare and community or public health settings; to include both PhD and MD respondents; and to include respondents with varying degrees of experience in (a) health service outcomes research, (b) implementation science in general, and (c) RE-AIM in particular. Because our interests from a systems science perspective were broad and included a large number of potential associations, we focused our primary analyses on several *a priori* hypotheses about which RE-AIM outcomes would be perceived as most strongly related to each service outcome.

The purpose of this paper is to report results of our on-line cross-sectional survey, including: [1] describing perceived relationships between RE-AIM implementation outcomes and service outcomes; [2] identifying which perceived relationships are strongest via tests of five *a priori* hypotheses; and [3] exploring potential differences in perceptions of relationships across several subgroups of participants categorized by discipline, setting, and levels of implementation science, health services, and RE-AIM experience.

Based on our team’s extensive experience with RE-AIM, implementation science more broadly, and health services research, our hypotheses were that among the RE-AIM outcomes,

### Hypothesis 1

Implementation/Fidelity would have the strongest perceived relationship with Effectiveness (as a service outcome). This hypothesis was based on the importance of intervention fidelity in traditional health intervention efficacy and effectiveness research.

### Hypothesis 2

Implementation/Cost would have the strongest perceived relationship with Efficiency. This hypothesis was based on the potential conceptual overlap between cost-effectiveness and efficiency.

### Hypothesis 3

Reach would have the strongest perceived relationship with Equity. The definition of Reach includes representativeness of those who receive an intervention compared to all who could benefit from it, and the equity implications of representativeness were the basis for this hypothesis.

### Hypothesis 4

Implementation/Adaptation would have the strongest perceived relationship with Patient-Centeredness. Given our team’s prior research on adaptations, we expected that the valuing of patient preferences as part of the adaptation process would relate to the principles of patient-centered care.

### Hypothesis 5

Implementation/Fidelity would have the strongest perceived relationship with Safety. This hypothesis was based on the definition of fidelity as delivery of an intervention as intended, including intervention integrity and quality of delivery. Its perceived relationship with safety was hypothesized due to the phased trials that evidence-based interventions are subject to, suggesting that when they are delivered with fidelity they should be safe, efficacious, and effective.

We did not hypothesize a specific RE-AIM outcome with the strongest perceived relationship with Timeliness.

## Methods

### Study design

We used a cross-sectional on-line survey study design to obtain data from a large convenience sample of respondents.

### Survey development

The on-line survey was developed in Qualtrics by the authors with attention to feasibility, conceptual clarity, and length using a “think-aloud” protocol [19] and pilot testing. Participants in the “think aloud” process (N = 4) included a public health researcher with administrative, teaching, and practice experience; 2 physician-researchers with health services research and implementation science expertise; and a public health graduate student. Participants who pilot tested final iterations of the survey (N = 14) included 10 doctoral-level researchers, 1 masters-level researcher, and 3 clinicians. Their primary research or practice settings of interest included primary care or outpatient settings; hospital or inpatient settings; communities or community-based organizations; and schools. All levels of experience with implementation science, health services research, and RE-AIM (see **Respondent Characteristics** below for categories) were represented in the pilot sample, each by multiple participants. Participants across the phases of survey development and piloting were not informed of study hypotheses. Survey content was finalized after several rounds of iterative revisions, yielding four sections: [1] respondent characteristics, [2] provision of introductory information to guide ratings, [3] establishing a reference scenario, and [4] ratings of perceived relationships between RE-AIM and service outcomes. The final survey is included as Additional File 1.

#### Respondent characteristics

The following respondent characteristics were assessed using close-ended multiple choice items with the option to write in “other” responses: current primary employment setting (*medical setting*, *public health or community organization*, *university*, or *other*); current primary professional role (*clinician*, *public health professional*, *researcher or educator*, *student*, or *other*); highest academic degree (*below bachelor’s degree*, *bachelor’s degree*, *master’s degree*, *PhD or DrPH*, *MD*, *other doctoral degree*, or *other degree*); year in which highest degree was earned; and primary research or practice setting of interest (*primary care or outpatient setting*, *hospital or inpatient setting*, *community or community-based organization*, *school*, *worksite*, *policy-making setting*, or *other*).

Levels of experience in (a) implementation science and (b) health service outcomes research were assessed by self-ratings (*a little or none*, *a moderate amount*, or *a lot*). Level of familiarity with the RE-AIM framework was assessed with an item briefly describing RE-AIM and asking for a self-rating (*have never heard of it, have heard of it but never used it before, have used it up to a few times, use it frequently*).

#### Introductory information

Because we aimed to include a broad range of health and public health researchers and practitioners, we included a brief introductory section including definitions of implementation outcomes and service outcomes, a version of Fig. 1 for reference, and an explanation of the purpose of the survey.

#### Reference scenario: initial and final versions

In the initial version, respondents were instructed to describe the implementation of a program, policy, or intervention that had happened in their setting in an open-ended text box. This was intended to ground respondents’ subsequent ratings of perceived relationships between RE-AIM and service outcomes with an actual implementation experience. In pilot testing, the wide variability in interpretation of this instruction and responses suggested that the goal of having all respondents engage in ratings based on a specific implementation experience was not met.

Through several iterations, the final reference scenario was designed to be more concrete and standardized. Rather than describing an implementation experience, respondents were asked to think about a hypothetical evidence-based program to increase adherence to recommended guidelines relevant to their setting, population, and area of research or practice. Examples of potential guidelines were provided to help respondents envision a program (e.g., guidelines for cancer screenings, promoting physical activity, limiting children’s screen time, etc.). The instructions reminded respondents to keep this example in mind when answering the next sections of the survey.

#### Rating perceived relationships: initial and final approaches

The initial survey was designed with the intent to measure perceived relationships between RE-AIM outcomes and service outcomes directionally (i.e., capturing the perceived strength of both positive and negative relationships between outcomes). The instructions prompted the respondent to think about the previously introduced implementation scenario, and then to rate each pairing of RE-AIM and service outcomes using a rating scale with response options from − 2 (*strong negative relationship*) to + 2 (*strong positive relationship*).

The “think aloud” process revealed that respondents struggled with the cognitive burden of thinking through the potential relationships between each pairing and to apply the original rating scale. This led us to simplify the item stems and limit the types of relationships respondents had to think through. The final version of items asked respondents to consider the likely changes they would observe in service outcomes as RE-AIM outcomes increased (i.e., “as this RE-AIM outcome goes up, what would you generally expect to observe about this service outcome?”). In parallel, response options were revised to be more concrete (i.e., *decreases a lot*, *decreases a little*, *no change*, *increases a little*, *increases a lot*).

Within the survey structure, pairs of outcomes to be rated were grouped by RE-AIM outcome. At the top of each set of ratings, a version of Fig. 1 was included to remind respondents of the theorized IOF connection between implementation, service, and health outcomes. The RE-AIM outcome for the specific section (e.g., Reach) was introduced and defined. Each IOM service outcome and its definition was then listed, and respondents selected a response option for each pairing (e.g., for Reach and Effectiveness, Reach and Efficiency, Reach and Equity, etc.). The order in which the service outcomes were listed was determined during pilot testing, with the “easiest” service outcomes to rate listed first. This strategy was recommended by pilot participants.

### Recruitment and survey administration

Recruitment involved four planned phases of enrolling a convenience sample, with a goal of survey completion by at least 200 respondents. The invitation schedule is shown in Table 2 by method, date range, and number of responses. The first round of survey administration was to our institutional colleagues (i.e., researchers and practitioners connected to our dissemination and implementation research program and broader research center). Next, our team identified and emailed professional contacts to request survey completion and dissemination of the invitation to others. The third round of recruitment and survey administration was through emails to national agencies, associations, and organizations requesting dissemination of the survey information and invitation, followed by a fourth round of dissemination via social media (Twitter). Receipt of invitations and survey progress were not able to be tracked per individual, so we did not have the ability to calculate response rates or send individual reminders to respondents.

**Table 2:** Responses by contact method and date range

Contact Method	Date Range	Number of Responses
Invitations to institutional colleagues	02/15/22 – 03/07/22	23
Emails to professional contacts	03/08/22 − 03/20/22	202
Emails to national association and organization listservs	03/21/22 − 03/30/22	105
Twitter	03/31/22 − 04/15/22	47
Total	02/15/22 – 04/15/22	377^a^

^a^Of 377 surveys submitted, 259 had complete data for ratings of RE-AIM and service outcomes and were used in analyses

The survey was administered via Qualtrics between February and April 2022, and responses were collected anonymously. Study information was provided on the first page of the survey, consent to participate was communicated via survey completion, and no compensation was provided to respondents. This project was reviewed and considered exempt by the Colorado Multiple Institutional Review Board.

### Analyses

Only surveys with complete data (N = 259) for ratings of perceived relationships between RE-AIM and service outcomes were included in analyses. Descriptive analyses summarized respondent characteristics, as well as mean ratings and standard deviations of perceived relationships between each pair of RE-AIM and service outcomes. All analyses were performed using IBM SPSS version 29. Because we planned five tests of hypotheses, a Bonferroni correction was applied in the primary analyses, adjusting the level of significance for each overall F statistic to *p* < .01 (0.05/5).

Perceived relationships between RE-AIM and service outcomes were analyzed using one-way repeated measures analysis of variance (RM ANOVA), testing ratings for service outcomes as the dependent variable and paired RE-AIM outcomes as the within-subjects independent variable. Assumptions for RM ANOVA were checked for each test. A Greenhouse-Geisser correction was applied in all RM ANOVAs to account for violations of the sphericity assumption. If the overall F statistic was statistically significant (*p* < .01 with Bonferroni adjustment), then pairwise comparisons using the Bonferroni post hoc test were used to identify which mean ratings differed, maintaining the family-wise *p* < .01 level of significance. The main analyses were directed by the five *a priori* hypotheses.

In addition to hypothesis testing, we planned two sets of exploratory analyses. First, we included Timeliness in the RM ANOVAs to explore what RE-AIM outcomes were perceived as most strongly related to this service outcome, in the absence of a hypothesis. Next, we used two-way RM ANOVAs to explore whether perceived relationships were moderated by four respondent characteristics (each tested separately): self-rated level of implementation science expertise (dichotomized into *a lot* versus *a little or none* and *a moderate amount*); self-rated level of health services research expertise (dichotomized into *a lot* versus *a little or none* and *a moderate amount*); self-rated level of familiarity with RE-AIM (dichotomized into *use it frequently* versus all others); and professional role (dichotomized into *clinicians* versus all others). Two respondents were missing data on at least one characteristic, reducing the sample size for these analyses by 1–2 (see Additional File 3, Appendix Table B). A significance level of *p* < .05 was used for all exploratory analyses.

## Results

### Respondent characteristics

Of the 377 surveys initiated, complete data for ratings of perceived relationships between RE-AIM and service outcomes were provided by 259 respondents and included in analyses. Most respondents were employed in university (55%) or medical settings (30%). The large majority of respondents were researchers or educators (78%), followed by clinicians (13%). Similarly, most had doctoral (61%) or medical (19%) degrees. When asked to rate their familiarity with the RE-AIM framework, 43% of respondents reported using RE-AIM up to a few times, while lower proportions reported using it frequently (29%), having heard of it but not used it (22%), or never having heard of it (6%). Distributions of experience with implementation science and health services research were similar, with approximately half of respondents reporting a moderate amount of experience (50% for implementation science, 43% for health services research), and lower proportions reporting little to no experience or a lot of experience. More details on respondent characteristics are presented in Table 3.

**Table 3:** Characteristics of respondents with complete ratings data (N = 259)

Variable	N (%)
Employment setting	
Medical setting	77 (30%)
Public health/community organization	16 (6%)
University	143 (55%)
Other ^a^	23 (9%)
Primary professional role^†^	
Clinician (e.g., physician, nurse, other HCP)	33 (13%)
Public health professional (e.g., health educator, admin)	8 (3%)
Researcher or educator	202 (78%)
Student	11 (4%)
Other ^b^	4 (2%)
Highest academic degree	
Bachelor’s degree	6 (2%)
Master’s degree	44 (17%)
Doctoral degree (PhD, DrPH, DNP, PsyD, etc.) ^c^	159 (61%)
MD ^c^	50 (19%)
Primary research/practice setting of interest	
Primary care or outpatient setting	121 (47%)
Hospital or inpatient setting	40 (15%)
Community or community-based organization	54 (21%)
School	13 (5%)
Worksite	5 (2%)
Policy-making setting	12 (5%)
Other ^d^	14 (5%)
Familiarity with RE-AIM framework	
Have never heard of it	16 (6%)
Have heard of it, but never used it before	57 (22%)
Have used it up to a few times	111 (43%)
Have used it frequently	75 (29%)
Implementation science experience	
A little or none	62 (24%)
A moderate amount	129 (50%)
A lot	68 (26%)
Health service outcomes research experience^†^	
A little or none	57 (22%)
A moderate amount	112 (44%)
A lot	89 (34%)

*Note*: Respondents ranged in years since earning their highest degree from 1 year to 56 years (M = 16 years, SD = 12 years)

^†^One missing response

^a^Other employment settings included government agencies, other research institutions, professional associations, and private companies

^b^Other primary professional roles included research administration and support and organization-specific roles

^c^Practice-oriented doctoral degrees (DNP, DrPH, PsyD) are included. Dual MD-PhD degrees are categorized with MD.

^d^Other settings of interest include health systems, health insurance, cross-sector, population-level, and general

### Descriptive analysis: perceived relationships of RE-AIM outcomes with service outcomes

**Fig. 2:**
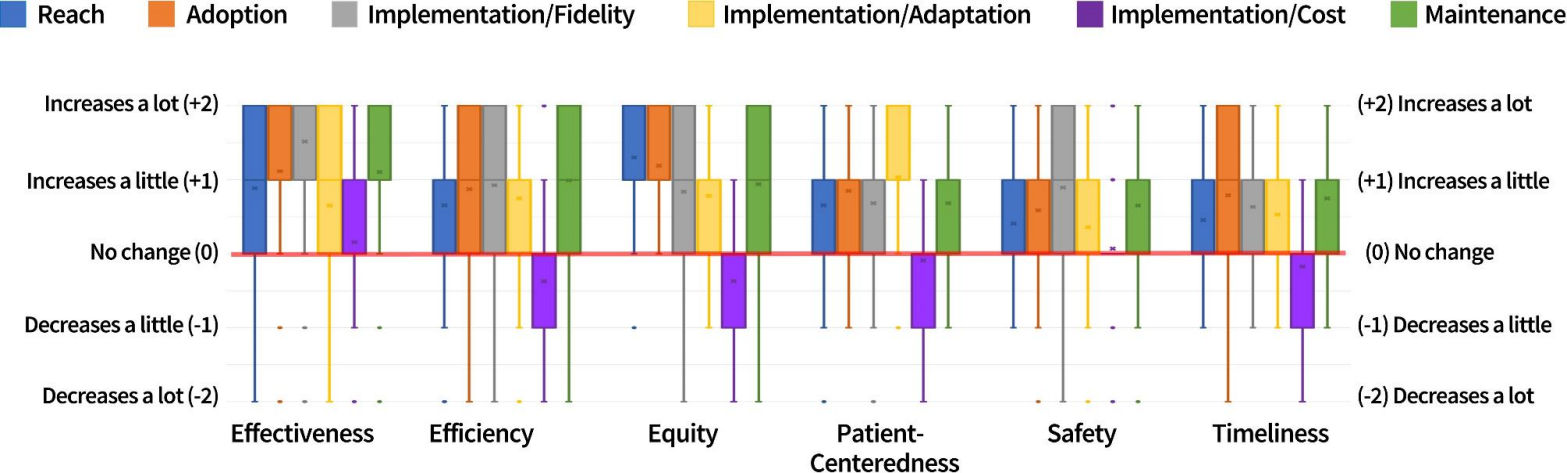
Box-and-whisker plots of mean ratings for each pairing of RE-AIM and service outcomes. Service outcomes are on the x-axis. Ratings of perceived relationships are on the y-axis. The red line highlights the rating of 0 (*No Change*). The “x” in each box indicates the mean. The lower and upper box edges indicate the 1st and 3rd quartiles. The whiskers extend to the minimum and maximum values within 1.5 times the interquartile range above or below the 1st and 3rd quartiles. Outliers appear as points outside that range

For each pairing, respondents were asked, “As the program is delivered, if [RE-AIM outcome] increases, what would you generally expect to observe about [service outcome]?” For analyses, we assigned the following values to the response options to facilitate interpretation: -2 = “decreases a lot,” -1 = “decreases a little,” 0 = “no change,” 1 = “increases a little,” and 2 = “increases a lot.” Means and standard deviations of ratings are reported in Table 4 and illustrated in Fig. 2. With these values, scores above 0 indicate a positive perceived relationship between the RE-AIM outcome and the service outcome; scores below 0 indicate a negative perceived relationship between the RE-AIM outcome and the service outcome; and scores near 0 indicate little to no perceived relationship between the RE-AIM outcome and the service outcome. Mean ratings ranged from a minimum of -0.37 (Implementation/Costs and Efficiency, Implementation/Costs and Equity) to a maximum of 1.52 (Implementation/Fidelity and Effectiveness). Standard deviations illustrated variability in ratings of each pairing, from the lowest standard deviation of 0.68 (Implementation/Fidelity and Effectiveness) to the highest standard deviation of 1.15 (Implementation/Adaptations and Effectiveness).

**Table 4:** Ratings of perceived relationships of RE-AIM outcomes with service outcomes (N = 259)

	Service Outcomes					
RE-AIM Outcomes ^a^	Effectiveness ^a^ M(SD)	Efficiency M(SD)	Equity M(SD)	Patient-Centeredness M(SD)	Safety M(SD)	Timeliness M(SD)
Reach	0.88 (1.04) ^b^	0.66 (1.00) ^b^	1.30 (0.81) ^b^	0.66 (0.98) ^b^	0.41 (0.93) ^b^	0.46 (1.06) ^b^
Adoption	1.12 (0.99) ^c^	0.87 (0.94)	1.19 (0.77) ^b^	0.85 (0.84) ^c^	0.59 (0.92) ^c^	0.79 (0.96) ^c^
Implementation/Fidelity	1.52 (0.68)	0.93 (0.98)	0.84 (0.94) ^c^	0.68 (0.95) ^bc^	0.90 (0.84)	0.64 (0.96) ^bc^
Implementation/Adaptation	0.65 (1.15) ^b^	0.75 (0.96) ^b^	0.78 (0.93) ^c^	1.03 (0.89) ^c^	0.36 (0.93) ^b^	0.53 (0.86) ^b^
Implementation/Cost	0.16 (0.96)	-0.37 (1.10)	-0.37 (1.09)	-0.09 (0.95)	0.07 (0.80)	-0.17 (1.03)
Maintenance	1.10 (0.96) ^c^	0.99 (0.89)	0.94 (0.85) ^c^	0.68 (0.88) ^bc^	0.65 (0.87) ^c^	0.75 (0.86) ^c^

*Note.* For each pairing, respondents were asked to select what they would generally expect to be observed in the service outcome if the RE-AIM outcome were to increase (e.g., “As the program is delivered, if REACH increases, what would you generally expect to observe about each service outcome: effectiveness, efficiency, equity, patient-centeredness, safety, timeliness”). Response options were assigned the following values: -2 = Decreases a lot; -1 = Decreases a little; 0 = No change; 1 = Increases a little; 2 = Increases a lot. Scores above 0 indicate a perceived positive relationship between the RE-AIM outcome and the service outcome; scores below 0 indicate a perceived negative relationship between the RE-AIM outcome and the service outcome; and scores near 0 indicate no perceived relationship between the RE-AIM outcome and the service outcome

^a^ Effectiveness is a RE-AIM outcome and a service outcome. The survey included Effectiveness as both. In analyses, Effectiveness is used only as a service outcome to decrease redundancy

^bc^ Within each column (service outcome), mean ratings that did *not* significantly differ from each other are signified using the same superscript letter. Significant pairwise comparisons had *p* < .01 using the Bonferroni post hoc test. See Additional File 2, Appendix Table A for *p-*values of comparisons

### Results of hypothesis-testing

For each service outcome, RM ANOVAs compared the mean ratings of each hypothesized RE-AIM outcome’s relationship with that service outcome (i.e., mean ratings were compared of the perceived relationships between Reach and Efficiency, Adoption and Efficiency, Implementation/Fidelity and Efficiency, Implementation/Adaptation and Efficiency, Implementation/Cost and Efficiency, and Maintenance and Efficiency). If the mean ratings were not equivalent (as indicated by the overall F test with Bonferroni-adjusted *p* < .01), post-hoc Bonferroni pairwise comparisons identified the pairings that differed significantly from others (*p* < .01). Our hypotheses specified which RE-AIM outcomes we expected to be most strongly related to each service outcome, and results are described below. Results of one-way RM ANOVAs are summarized in Table 5, and results of post-hoc pairwise comparisons among RE-AIM outcomes are noted in Table 4. Additional File 2 includes Appendix Table A reporting *p*-values for post-hoc pairwise comparisons.

**Table 5:** One-Way repeated measures analyses of variance in perceived relationships of service outcomes with RE-AIM outcomes (N = 259)

Service Outcome	F(*df1, df2*)	η^2^
Effectiveness	F(4.16, 1068.93) = 71.91***	0.22
Efficiency	F(4.39, 1124.50) = 81.91***	0.22
Equity	F(3.98, 1022.85) = 138.41***	0.32
Patient-Centeredness	F(4.10, 1055.09) = 60.85***	0.19
Safety	F(4.39, 1128.88) = 37.08***	0.13
Timeliness	F(4.16, 1068.54) = 44.18***	0.14

*Note*: Degrees of freedom and test statistics are based on the Greenhouse-Geisser correction for sphericity. Means and standard deviations of ratings are reported in Table 3 and not repeated here

^***^p < .001

#### RE-AIM outcomes and effectiveness (as a service outcome)

Mean ratings for perceived relationships of each RE-AIM outcome with the service outcome Effectiveness differed significantly, F(4.16, 1068.93) = 71.91, *p* < .001. Post-hoc pairwise comparison revealed that all mean RE-AIM outcome ratings were significantly different from each other (*p* < .01) except for Reach versus Implementation/Adaptation (*p* = .13), Reach versus Maintenance (*p* = .04), and Adoption versus Maintenance (*p* = .99). Implementation/Fidelity had the strongest perceived relationship with Effectiveness (M = 1.52, SD = 0.68), supporting Hypothesis 1.

#### RE-AIM outcomes and efficiency

Mean ratings for perceived relationships of each RE-AIM outcome with the service outcome Efficiency varied significantly, F(4.39, 1124.50) = 81.91, *p* < .001. Post-hoc tests revealed that all mean RE-AIM outcome ratings were significantly different from each other (*p* < .01) except for Reach versus Implementation/Adaptation (*p* = .99); Adoption versus Implementation/Fidelity, Implementation/Adaptation, and Maintenance (all *p* = .99); Implementation/Fidelity versus Implementation/Adaptation (*p* = .54) and Maintenance (*p* = .99); and Implementation/Adaptation versus Maintenance (*p* = .04). Maintenance had the strongest perceived relationship with Efficiency (M = 0.99, SD = 0.89). Hypothesis 2 was that the strongest perceived relationship with Efficiency would be Implementation/Cost, and this hypothesis was not supported.

#### RE-AIM outcomes and equity

Mean ratings for perceived relationships of each RE-AIM outcome with the service outcome Equity differed significantly, F(3.98, 1022.85) = 138.41, *p* < .001. Post-hoc tests revealed that all mean RE-AIM outcome ratings were significantly different from each other (*p* < .01) except for Reach and Adoption (*p* = .48), and for Implementation/Fidelity, Implementation/Adaptation, and Maintenance (*p* = .44 and 0.99). Reach had the strongest perceived relationship with Equity (M = 1.30, SD = 0.81), closely followed by Adoption and Equity (M = 1.19, SD = 1.77). Hypothesis 3 was that Reach would have the strongest perceived relationship with Equity. This hypothesis was supported, though the perceived relationship between Adoption and Equity was similarly strong.

#### RE-AIM outcomes and patient-centeredness

Mean ratings for perceived relationships of each RE-AIM outcome with the service outcome Patient-Centeredness differed significantly, F(4.10, 1055.09) = 60.85, *p* < .001. Post-hoc pairwise comparisons revealed that all mean RE-AIM outcome ratings were significantly different from each other (*p* < .01) except for Reach versus Adoption (*p* = .02); Reach, Implementation/Fidelity, and Maintenance (*p* = .99); Adoption versus Implementation/Fidelity (*p* = .08), Implementation/Adaptation (*p* = .13), and Maintenance (*p* = .05); and Implementation/Fidelity versus Maintenance (*p* = .99). Implementation/Adaptation had the strongest perceived relationship with Patient-Centeredness (M = 1.03, SD = 0.89), followed by Adoption and Patient-Centeredness (M = 0.85, SD = 0.84). Hypothesis 4 was that Implementation/Adaptation would have the strongest perceived relationship with Patient-Centeredness. This hypothesis was also supported, with the addition of Adoption also having a similarly strong perceived relationship with Patient-Centeredness compared to the other RE-AIM outcomes.

#### RE-AIM outcomes and safety

Mean ratings for perceived relationships of each RE-AIM outcome with the service outcome Safety differed significantly, F(4.39, 1128.88) = 37.08, *p* < .001. Post-hoc tests revealed that all mean ratings for pairs of RE-AIM outcomes differed significantly in their ratings (*p* < .01) except for Reach versus Adoption (*p* = .03) and Implementation/Adaptation (*p* = .99); and Adoption versus Implementation/Adaptation (*p* = .01) and Maintenance (*p* = .99). Implementation/Fidelity had the strongest perceived relationship with Safety (M = 0.90, SD = 0.84), supporting Hypothesis 5.

### Results of exploratory analyses

#### RE-AIM outcomes and timeliness

Parallel to results of RM ANOVAs testing our hypotheses, the mean ratings for perceived relationships of each RE-AIM outcome with the service outcome Timeliness differed significantly, F(4.16, 1068.54) = 81.91, *p* < .001. Post-hoc tests revealed two groups of RE-AIM outcomes within which mean ratings did not significantly differ: Reach, Implementation/Fidelity, and Implementation/Adaptation (*p* = .20 and 0.99); and Adoption, Implementation/Fidelity, and Maintenance (*p* = .32 and 0.99). Three RE-AIM outcomes had the strongest perceived relationships with Timeliness: Adoption (M = 0.79, SD = 0.96), Implementation/Fidelity (M = 0.64, SD = 0.96), and Maintenance (M = 0.75, SD = 0.87). Compared to the strength of perceived relationships of RE-AIM outcomes with the other service outcomes in hypothesis testing analyses, ratings of relationships with Timeliness were relatively weaker.

#### Differences in ratings among subgroups of respondents

Results of four separate two-way RM ANOVAs revealed no significant differences in perceived relationships between RE-AIM outcomes and service outcomes among subgroups of respondents. Mean ratings were not found to differ between groups dichotomized by self-rated level of implementation science expertise, F(3.98, 1017.97) = 0.95, *p* = .43; self-rated level of health services research expertise, F(3.99, 1018.06) = 0.40, *p* = .81; self-rated level of familiarity with RE-AIM, F(3.99, 1020.52) = 1.65, *p* = .16; or professional role, F(3.98, 1019.24) = 1.47, *p* = .21. Means and standard deviations for all subgroups are reported in Additional File 3, Appendix Table B.

## Discussion

Greater specification and understanding of the links among implementation outcomes and service outcomes [6] will advance D&I science by clarifying pathways from implementation and sustainment of interventions to population health outcomes [20]. It is important to understand different factors involved in the chain of events from implementation to service outcomes to eventual population health outcomes—and to evaluate potential unintended consequences [21, 22]. These relationships have been understudied, and service outcomes are rarely included in implementation studies. To our knowledge this is the first investigation of the relationship of implementation outcomes to service outcomes, with a focus on perceptions of these relationships among respondents with varying levels of experience with implementation science, health services research, and the RE-AIM framework.

The majority of our *a priori* hypotheses about the perceived relationships of different RE-AIM outcomes with service outcomes were supported. We were especially interested in which RE-AIM outcomes were perceived as most strongly related to health equity outcomes. As predicted in Hypothesis 3, Reach was the RE-AIM dimension perceived to be most strongly related to Equity: the mean rating of this relationship was also one of the strongest in the study. In addition, Adoption was also perceived as strongly related to Equity. While not included in our original hypotheses (each limited to a single RE-AIM outcome expected to have the strongest relationship), this result was not surprising: inequitable (or non-representative) adoption of evidence-based programs by settings and staff serving those most in need leads to limited access, lack of equitable benefit, and inequity [23, 24].

As predicted in Hypotheses 1 and 5, Implementation/Fidelity was clearly the RE-AIM factor perceived as most strongly related to both Effectiveness and Safety. This was expected, but in designing our exploratory analyses, we had wondered whether respondents with more implementation science experience would perceive Implementation/Adaptation as more strongly related to Effectiveness, compared to ratings by those with less experience. The implementation science emphasis on the importance of adaptation for successful implementation and sustainment by increasing the “fit” of an intervention with recipients, deliverers, organizations, and systems suggests that Implementation/Adaptation may also improve Effectiveness (e.g., cultural adaptations of evidence-based psychological interventions) [25, 26]. This potential difference in ratings based on implementation science experience was not observed, however.

We were interested to find that one of our more nuanced predictions was also supported: Hypothesis 4, that Implementation/Adaptation would be perceived as strongly related to Patient-Centeredness. This suggests that respondents generally conceptualized tailoring or adaptation as reflecting greater sensitivity to the patient’s situation, a key aspect of patient-centered care. Perceived relationships between Implementation/Adaptation and the other service outcomes, however, were weaker. Effectiveness, Efficiency, and Equity, in particular, are service outcomes that seem aligned with the intended effects of adapting evidence-based programs for new contexts [27–29], but were not rated here as having strong perceived relationships with Implementation/Adaptation. Given the challenges encountered in the survey design phase to provide adequate information yet not overwhelm respondents in a cognitively demanding task, it is possible that ratings of the relationships between Implementation/Adaptation and service outcomes were close to 0 because of the wide variety of potential adaptations that could be and are made in practice. Some adaptations may have positive relationships with service outcomes, some negative, and some may have no relationship at all [28]. Additional items specifying different types of adaptations (e.g., guided by organizational tools like the FRAME [30]) would likely be necessary to further clarify perceptions of these relationships.

Hypothesis 2 was the only prediction to receive no support: Implementation/Cost was not the RE-AIM outcome perceived as most strongly related to Efficiency. There are multiple possible explanations of this finding. It could be that respondents did not adequately understand the definitions of these outcomes; that some respondents may not fully appreciate the potential impact of implementation costs on outcomes; that the directionality implied by the wording of items on the survey confused raters; or that the relationship between these outcomes may vary depending on the situation, leading to variable ratings. Among the RE-AIM outcomes, only Implementation/Cost had negative mean ratings of perceived relationship with service outcomes, including Efficiency (M = -0.37), Equity (M = -0.37), Patient-Centeredness (M = -0.09), and Timeliness (M = -0.17). Given the directionality of item wording, this means that respondents generally expected these service outcomes to worsen when costs increased. When mean ratings for Implementation/Cost were positive (i.e., perceived relationships with Effectiveness and Safety), they were also quite close to zero—suggesting that this RE-AIM outcome may have been difficult for respondents to rate or more complicated than its definition in the survey could accommodate.

To place these results in context, development of the survey required several challenging (and sometimes compromising) decisions. We wanted respondents to think through positive and negative relationships between RE-AIM and service outcomes, as well as to consider bidirectional effects (i.e., that service outcomes could influence RE-AIM outcomes). However, when the initial survey format and items were constructed to encompass these possibilities, participants in survey development and pilot testing were confused and overwhelmed thinking through the implications, and survey completion became time-consuming and frustrating. Additionally, we wanted to include a variety of respondents with varied familiarity with implementation science, health services research, and RE-AIM. This required inclusion of introductory material and many definitions, increasing the burden of survey completion. Despite considerable pilot testing and use of survey development processes like the “think aloud” technique [19], some of our brief definitions of RE-AIM and service outcomes may have been difficult to understand for respondents with less experience or familiarity with implementation science and RE-AIM, or conversely, too restricted for respondents with high levels of experience. For example, Implementation/Adaptation in implementation science is typically viewed as a “given” and as improving the fit of programs to specific contexts, but outside of implementation science, adaptations can be viewed as negative, posing threats to fidelity. The quality, type, and extent of adaptations may be important to know in trying to rate their relationships with all service outcomes, and this was challenging to present in a survey format while limiting participant burden. As described above, similar challenges were observed with ratings of the relationships of Implementation/Cost with each service outcome.

Overall, however, we believe that our survey development decisions facilitated a pragmatic and much needed initial appraisal of the perceived relationships among implementation and service outcomes. The similarity of findings across respondent characteristics including professional discipline, level of experience in health service outcomes research, level of experience in implementation science, and degree of familiarity with RE-AIM suggests a somewhat surprising consistency with which a wide variety of U.S. health services and implementation researchers view the impacts of program implementation. Results highlight potential relationships among RE-AIM and service outcomes that can eventually be tested with objective data and in meta-analyses. They also highlight important nuances in three implementation outcomes—Implementation/Fidelity, Implementation/Adaptation, and Implementation/Cost—that require further methodological exploration to refine their definitions and application in future studies.

### Limitations and strengths

There are several qualifications of our results. First, the relationships described were of respondent perceptions of how implementation outcomes are related to service outcomes, rather than analyses of actual empirical data on these outcomes across studies. Thus, we were essentially studying respondents’ mental models of the way implementation and service outcomes relate to each other, rather than “reality.” Given the lack of reporting of joint reporting of RE-AIM and service outcomes in empirical studies, it is currently not possible to conduct a review of previous studies. As described in our Methods section, our survey development process led us to frame relationships quite concretely so that respondents could rate them without confusion. While we carefully avoided language implying causal direction between RE-AIM outcomes and service outcomes, it is still possible that the framing affected ratings. There certainly could be inverse or reciprocal relationships in which service outcomes affect RE-AIM outcomes, and our study did not address these issues. The selection of a 5-point Likert-type response scale was based on feedback from participants in the pilot phase, who reported that the high level of complexity inherent in making these ratings required a somewhat simplified rating structure. It is possible that different response scales could yield different results. Additionally, the *no change* point on the Likert-type scale for rating pairings was intended to reflect no relationship between outcomes, but could have been used by respondents recognizing that “it depends” and that the relationship between outcomes could vary depending on other factors. The nature of perceived relationships (e.g., causal, correlational) were not addressed in this study, but should be explored in future research. Finally, our participant recruitment and online data collection strategies prevented us from being able to calculate response rates or assess the representativeness of respondents compared to all health services and public health researchers and practitioners who could have completed the survey.

There are also several strengths to this study, especially given that it is the first such investigation of this understudied issue. We conducted user testing and made iterative improvements in survey design that were pilot tested before launching the final survey. Although not a random sample, the number of respondents was relatively large. The diverse sample permitted investigation of potential differences across subgroups that we had identified as of interest prior to launching the study. We made a substantial effort to recruit participants from different disciplines and settings relevant to health and public health research and practice, especially with varying degrees of experience with health service outcomes research, implementation science, and the RE-AIM framework. Finally, our inclusion of *a priori* hypotheses and use of stringent Bonferroni corrections for multiple comparisons were methodological strengths.

### Conclusions and future directions

Proctor’s IOF [6] has served as a logic model of sorts to the field of implementation science since its publication. The actual relationships between implementation outcomes, service outcomes, and health and population health outcomes, however, have not been tested. Most implementation science studies, including those applying the IOF, do not incorporate service outcomes in their design, measures, or analyses. Health services researchers and others addressing health care quality often assess service outcomes as indicators of success. Implementation science researchers, in contrast, focus on implementation outcomes. All of these fields of research ultimately aim to improve health outcomes and population health. When multiple fields pursue similar end goals but with different interim definitions of success, it is crucial to understand when those outcomes may be complementary, synergistic, opposed, or unrelated.

To advance our understanding of these issues, in addition to replicating these findings in different samples and with different methods, researchers can contribute by consistently including data on service outcomes in their implementation studies. Eventually this would permit systematic reviews and meta-analyses to evaluate the generalizability of findings. Other studies could investigate the “reverse order” of perceived relationships—i.e., “when [service outcome] increases, what would you generally expect to observed of [RE-AIM outcome]”—and how directionality, response options, and other elements of survey design affect perceptions. Our preliminary findings suggest some predicted pathways between RE-AIM implementation outcomes and service outcomes and highlight areas for future research, including exploration of the types of relationships (perceived or actual) between outcomes (e.g., causal, correlational). It would be especially informative to study the “boundary conditions,” or the range of contextual factors that might impact both ratings of perceived and actual relationships, once necessary data are available. Regardless of the specific methods and conceptual models investigated, further investigations of relationships among different types of outcomes are needed both to advance implementation science and health services research, and to capitalize on their connections to ultimately improve population health.

### Electronic supplementary material

Below is the link to the electronic supplementary material.

Supplementary Material 1: Final survey

Supplementary Material 2: Appendix Table A, P-values > .001 for pairwise comparisons of mean ratings of perceived relationships of RE-AIM outcomes with service outcomes (N = 259)

Supplementary Material 3: Appendix Table B, ratings of perceived relationships of RE-AIM outcomes with service outcomes by respondent subgroups

## Data Availability

The dataset used in the current study is available from the corresponding author on reasonable request.

## Abbreviations

RE-AIM: Reach, Effectiveness, Adoption, Implementation, and Maintenance
IOF: Implementation Outcomes Framework
IOM: Institute of Medicine
RM ANOVA: Repeated measures analysis of variance
