# Supplementary material for: RE-AIM implementation outcomes and service outcomes: what’s the connection? results of a cross-sectional survey
Source: BMC Health Serv Res. 2023 Dec 15;23:1417. doi: 10.1186/s12913-023-10422-w (PMC10722784; doi:10.1186/s12913-023-10422-w)
Supplement: Supplementary file 1 — Supplementary Material 1: Final survey [file 12913_2023_10422_MOESM1_ESM.pdf]

University of Colorado **Anschutz Medical Campus**

Consent & Background

Relating Implementation to Quality Outcomes

We would like to understand your thoughts about the relationships among different kinds of outcomes that are important in health services and health-related research. You are being asked to be in this research study because you are either a health services researcher, health care provider or public health professional. If you join the study, you will take a survey lasting approximately 10-15 minutes. No identifying information is requested, and your responses will be confidential. Results will be reported only in summary fashion.

This survey is being conducted as part of the Pragmatic Implementation Science Project funded by The National Cancer Institute (P50CA244688). The research study is based at the University of Colorado | Anschutz Medical Campus. Participation in this survey is completely voluntary. By answering the questions and submitting the questionnaire you are providing consent and permission to use the information shared. We believe this research study presents no risk to all participants; if you do not want to respond to any question, simply skip it. There may be risks of which the researchers have not thought. This study is not designed to benefit you directly. The data we collect will be used for this study, but may also be important for future research. Your data may be used for future research or distributed to other researchers for future study without additional consent if information that identifies you is removed from the data. If you have questions, you can contact Bryan Ford at bryan.ford@cuanschutz.edu or 303.724.3422. If you have questions about your rights as a participant, you can call the COMIRB (the responsible Institutional Review Board) at 303-724-1055.

ATTENTION - We highly suggest completing this survey on your computer. (The mobile version is cumbersome)

Thank you for agreeing to complete this survey. To help us understand your background and your experiences with RE-AIM, please answer the following 5 questions

☐ I'm not a robot
reCAPTCHA
Privacy - Terms

What is your current, **primary** employment setting?

- ○ Medical Setting
- ○ Public health or community organization

- ◯ University
- ◯ [ ] Other (please specifiy)

What is your current, **primary** professional role?

- ◯ Clinician (e.g. physician, nurse, physician's assistant, medical student, other health care professional)
- ◯ Public health professional (e.g. health educator, administrator)
- ◯ Researcher or educator
- ◯ Student
- ◯ [ ] Other (please specify)

Please select your **highest** degree

- ◯ Below Bachelor's Degree
- ◯ Bachelor's degree
- ◯ [ ] Master's Degree (please specify)
- ◯ PhD or DrPH
- ◯ MD
- ◯ [ ] Other Doctoral degree (please specify)
- ◯ [ ] Other degree (please specify)

In what year did you **earn** your highest degree?

	1940	1948	1956	1964	1972	1981	1989	1997	2005	2013	2021	
Move the slider to indicate year												[ ]

How much experience do you have in health service outcomes research?

- ◯ A little or none
- ◯ A moderate amount
- ◯ A lot

How much experience do you have in implementation science?

- ◯ A little or none

- A moderate amount
- A lot

What is your primary research/practice setting of interest?

- Primary Care or Outpatient Setting
- Hospital or Inpatient Setting
- Community or Community-based Organization
- School
- Worksite
- Policy-making Setting
- [ ] Other (Please specify)

RE-AIM (Reach, Effectiveness, Adoption, Implementation, Maintenance) is a framework used for planning, implementing, and evaluating programs to improve population health. How familiar are you with the RE-AIM framework?

- Have never heard of it
- Have heard of it, but never used it before
- Have used it up to a few times
- Use it frequently

Intro

As shown in the figure below, when an evidence-based program is implemented using specific strategies (e.g., training staff, audit and feedback), three types of outcomes follow: implementation outcomes, quality/service outcomes, and health outcomes. However, little is known about the relationships among these types of outcomes.

In this survey, we will ask about your perceptions of the relationships among five **implementation outcomes** (reach, effectiveness, adoption, implementation, and maintenance) and the six **quality/service outcomes** defined by the Institute of Medicine in 2001 (safety, equity, efficiency, effectiveness, timeliness, patient-centeredness), circled in the figure below.

In answering these questions, please think about these implementation outcomes and quality/service outcomes in YOUR primary research/practice setting.

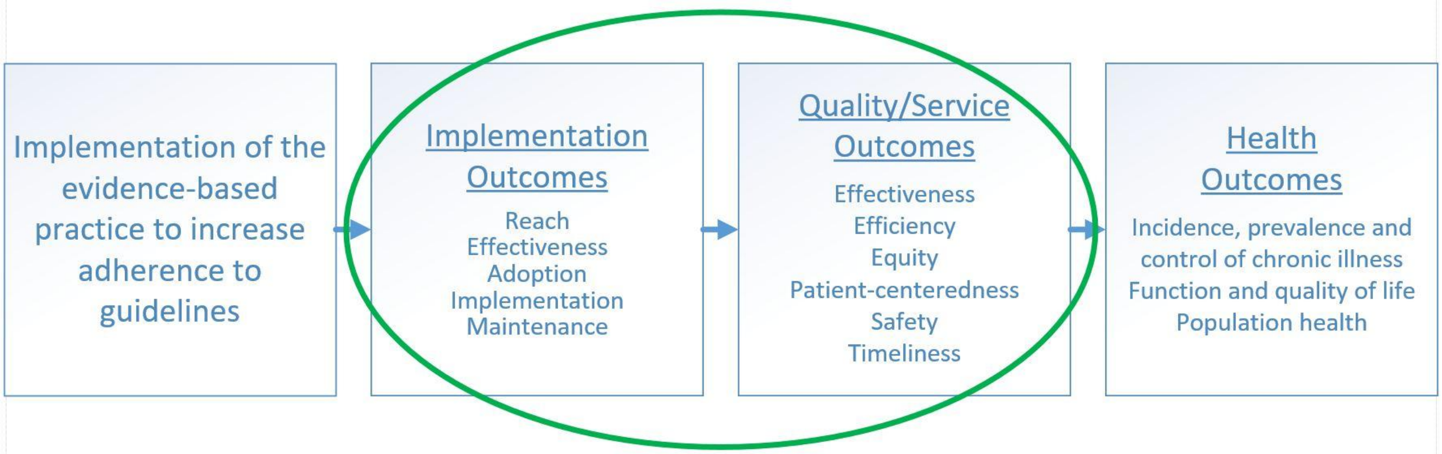

Think about a hypothetical *evidence-based program to increase adherence to recommended guidelines*. This could be one that you have implemented or one you'd like to implement in the future. The guidelines could include guidelines for cancer screenings, promoting physical activity, limiting young children's screen time, delivering smoking cessation counseling, conducting shared decision-making around vaccinations, etc. Think about a guideline related to your setting and the population with which you work. **Keep this ONE example in mind when answering each section of the survey.**

On each page of the survey, please review the provided definitions and then respond to all items, providing your best guess if you are not certain of the answer. There are no "right" answers! Please remember to use the entire scale.

Reach

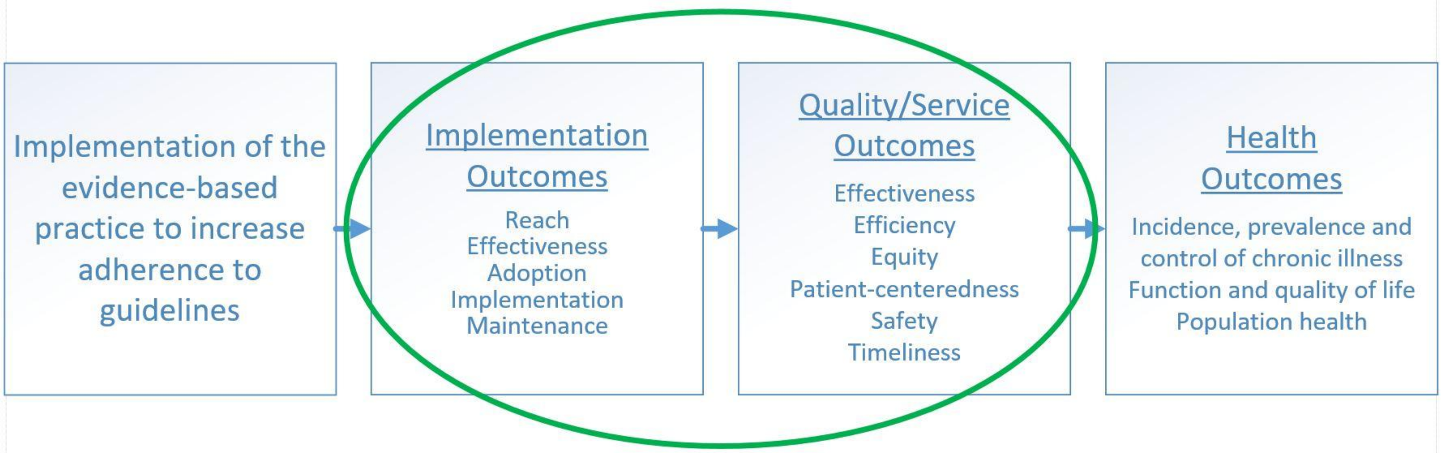

Reach

The first set of questions are about how the implementation outcome, REACH of the guideline adherence program, may be related to each quality/service outcome. **REACH is defined as the proportion and representativeness of individuals in the target population who receive the evidence-based program.**

In less technical language, **WHO** is intended to benefit and who actually participates or is exposed to the intervention?

Think about this evidence-based program being *delivered in your setting*. As the program is delivered, **if <u>REACH increases</u>, what would you generally expect to observe about each of the quality/service outcomes?** (be sure to use the entire scale):

	Decreases A Lot	Decreases A Little	No Change	Increases A Little	Increases A Lot
**EFFECTIVENESS?** (*Definition*: Providing care that is based on systematically acquired evidence demonstrating that a program or intervention produces better outcomes than alternatives—including the alternative of doing nothing.)	○	○	○	○	○
**EFFICIENCY?** (*Definition*: Avoiding waste, including waste of equipment, supplies, ideas, and energy.)	○	○	○	○	○
**EQUITY?** (*Definition*: Providing care that does not vary in quality because of personal characteristics such as gender, ethnicity, geographic location, and socioeconomic status.)	○	○	○	○	○
**PATIENT-CENTEREDNESS?** (*Definition*: Providing care that is respectful of and responsive to individual patient preferences, needs, and values and ensuring that patient values guide all clinical decisions.)	○	○	○	○	○
**SAFETY?** (Definition: Avoiding injuries to patients from the care that is intended to help them)	○	○	○	○	○
**TIMELINESS?** (*Definition*: Reducing waits and sometimes harmful delays for both those who receive and those who give care.)	○	○	○	○	○

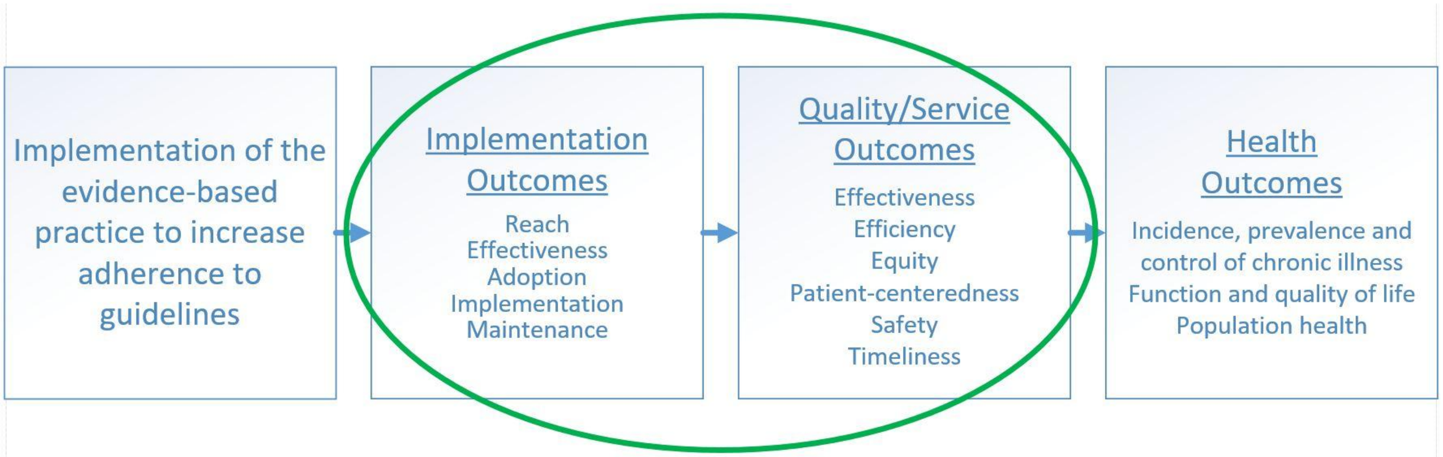

Effectiveness

The next set of questions are about how the implementation outcome, EFFECTIVENESS of the guideline adherence program, may be related to each quality/service outcome. **EFFECTIVENESS is defined as the *impact* on important outcomes of the evidence-based program aimed at increasing adherence to recommended guidelines. This includes potential negative effects as well as consistency of outcomes across groups.**

In less technical language, **DOES** the program work as intended, and what is the likelihood of negative outcomes?

Think about this evidence-based program being delivered in your setting. As the program is delivered, **if EFFECTIVENESS increases, what would you generally expect to observe about each of the quality/service outcomes?** (be sure to use the entire scale):

	Decreases A Lot	Decreases A Little	No Change	Increases A Little	Increases A Lot
**EFFICIENCY?** (*Definition*: Avoiding waste, including waste of equipment, supplies, ideas, and energy.)	○	○	○	○	○
**EQUITY?** (*Definition*: Providing care that does not vary in quality because of personal characteristics such as gender, ethnicity, geographic location, and socioeconomic status.)	○	○	○	○	○

	Decreases A Lot	Decreases A Little	No Change	Increases A Little	Increases A Lot
**PATIENT-CENTEREDNESS?** *(Definition*: Providing care that is respectful of and responsive to individual patient preferences, needs, and values and ensuring that patient values guide all clinical decisions.)	○	○	○	○	○
**SAFETY?** (Definition: Avoiding injuries to patients from the care that is intended to help them)	○	○	○	○	○
**TIMELINESS?** (*Definition*: Reducing waits and sometimes harmful delays for both those who receive and those who give care.)	○	○	○	○	○

Adoption

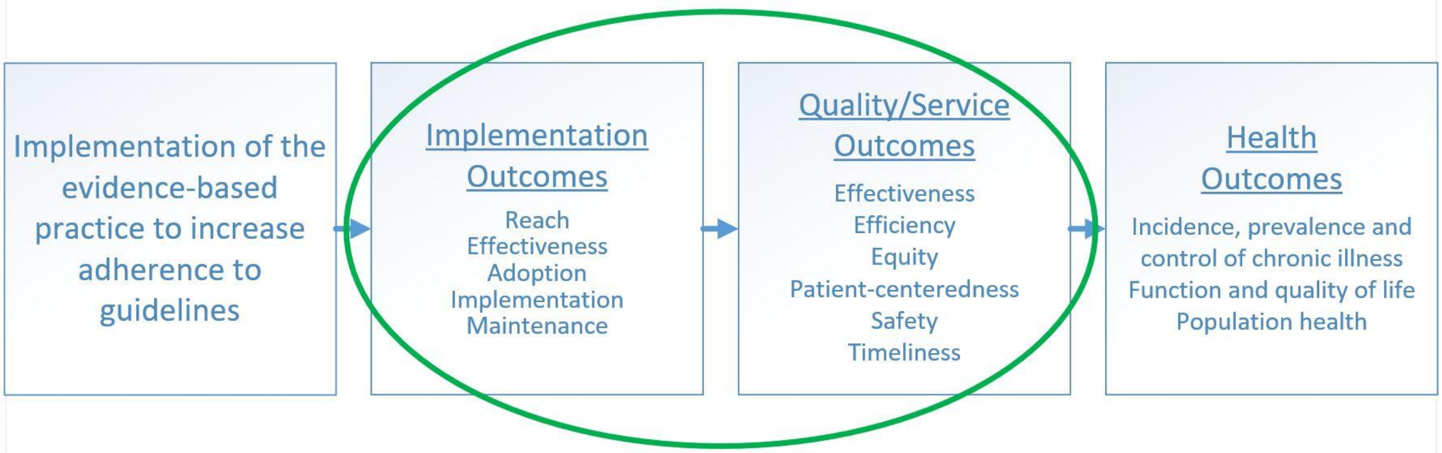

Adoption

The next set of questions are about how the implementation outcome, ADOPTION of the guideline adherence program, may be related to each quality/service outcome. **ADOPTION is defined as the proportion and representativeness of settings and staff that adopt the evidence-based program aimed at increasing adherence to recommended guidelines.**

In less technical language, **WHICH** settings and staff are intended to use the program, and how many actually use it?

Think about this evidence-based program being delivered in your setting. As the program is delivered, **if ADOPTION increases, what would you generally expect to observe about each of the quality/service outcomes?** (be sure to use the entire scale):

	Decreases A Lot	Decreases A Little	No Change	Increases A Little	Increases A Lot
**EFFECTIVENESS?** (*Definition*: Providing care that is based on systematically acquired evidence demonstrating that a program or intervention produces better outcomes than alternatives—including the alternative of doing nothing.)	○	○	○	○	○
**EFFICIENCY?** (*Definition*: Avoiding waste, including waste of equipment, supplies, ideas, and energy.)	○	○	○	○	○
**EQUITY?** (*Definition*: Providing care that does not vary in quality because of personal characteristics such as gender, ethnicity, geographic location, and socioeconomic status.)	○	○	○	○	○
**PATIENT-CENTEREDNESS?** *(Definition*: Providing care that is respectful of and responsive to individual patient preferences, needs, and values and ensuring that patient values guide all clinical decisions.)	○	○	○	○	○
**SAFETY?** (Definition: Avoiding injuries to patients from the care that is intended to help them)	○	○	○	○	○
**TIMELINESS?** (*Definition*: Reducing waits and sometimes harmful delays for both those who receive and those who give care.)	○	○	○	○	○

Implementation

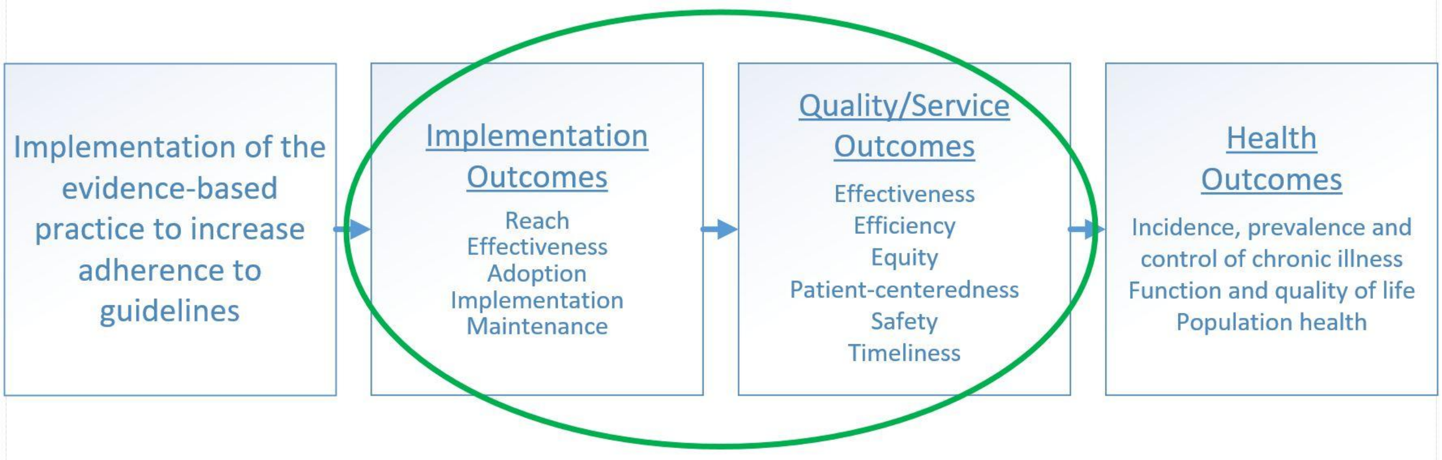

Implementation

The next <u>three</u> sets of questions are about how the implementation outcome, IMPLEMENTATION of the guideline adherence program, may be related to each quality/service outcome. **IMPLEMENTATION** of the evidence-based program aimed at increasing adherence to recommended guidelines has three components.

The first component of IMPLEMENTATION is **FIDELITY**, **defined as how consistently the evidence-based program is delivered as intended.**

Think about this evidence-based program being delivered in your setting. As the program is delivered, **if FIDELITY increases, what would you generally expect to observe about each of the quality/service outcomes?** (be sure to use the entire scale):

	Decreases A Lot	Decreases A Little	No Change	Increases A Little	Increases A Lot
**EFFECTIVENESS?** (*Definition*: Providing care that is based on systematically acquired evidence demonstrating that a program or intervention produces better outcomes than alternatives—including the alternative of doing nothing.)	○	○	○	○	○
**EFFICIENCY?** (*Definition*: Avoiding waste, including waste of equipment, supplies, ideas, and energy.)	○	○	○	○	○
**EQUITY?** (*Definition*: Providing care that does not vary in quality because of personal characteristics such as gender, ethnicity, geographic location, and socioeconomic status.)	○	○	○	○	○

	Decreases A Lot	Decreases A Little	No Change	Increases A Little	Increases A Lot
**PATIENT-CENTEREDNESS?** *(Definition*: Providing care that is respectful of and responsive to individual patient preferences, needs, and values and ensuring that patient values guide all clinical decisions.)	○	○	○	○	○
**SAFETY?** (Definition: Avoiding injuries to patients from the care that is intended to help them)	○	○	○	○	○
**TIMELINESS?** (*Definition*: Reducing waits and sometimes harmful delays for both those who receive and those who give care.)	○	○	○	○	○

The second component of IMPLEMENTATION is **ADAPTATIONS**, **defined as whether changes or modifications were made to the evidence-based program.**

Think about this evidence-based program being delivered in your setting. As the program is delivered, **if ADAPTATIONS increase, what would you generally expect to observe about each of the quality/service outcomes?** (be sure to use the entire scale):

	Decreases A Lot	Decreases A Little	No Change	Increases A Little	Increases A Lot
**EFFECTIVENESS?** (*Definition*: Providing care that is based on systematically acquired evidence demonstrating that a program or intervention produces better outcomes than alternatives—including the alternative of doing nothing.)	○	○	○	○	○
**EFFICIENCY?** (*Definition*: Avoiding waste, including waste of equipment, supplies, ideas, and energy.)	○	○	○	○	○

	Decreases A Lot	Decreases A Little	No Change	Increases A Little	Increases A Lot
**EQUITY?** (*Definition*: Providing care that does not vary in quality because of personal characteristics such as gender, ethnicity, geographic location, and socioeconomic status.)	○	○	○	○	○
**PATIENT-CENTEREDNESS?** (*Definition*: Providing care that is respectful of and responsive to individual patient preferences, needs, and values and ensuring that patient values guide all clinical decisions.)	○	○	○	○	○
**SAFETY?** (Definition: Avoiding injuries to patients from the care that is intended to help them)	○	○	○	○	○
**TIMELINESS?** (*Definition*: Reducing waits and sometimes harmful delays for both those who receive and those who give care.)	○	○	○	○	○

The third component of IMPLEMENTATION is **COSTS, defined as how much it costs to deliver the program, including expenses for personnel, materials, training and supervision.**

Think about this evidence-based program being delivered in your setting. As the program is delivered, **if COSTS increase, what would you generally expect to observe about each of the quality/service outcomes?** (be sure to use the entire scale):

	Decreases A Lot	Decreases A Little	No Change	Increases A Little	Increases A Lot
**EFFECTIVENESS?** (*Definition*: Providing care that is based on systematically acquired evidence demonstrating that a program or intervention produces better outcomes than alternatives—including the alternative of doing nothing.)	○	○	○	○	○

	Decreases A Lot	Decreases A Little	No Change	Increases A Little	Increases A Lot
**EFFICIENCY?** (*Definition*: Avoiding waste, including waste of equipment, supplies, ideas, and energy.)	○	○	○	○	○
**EQUITY?** (*Definition*: Providing care that does not vary in quality because of personal characteristics such as gender, ethnicity, geographic location, and socioeconomic status.)	○	○	○	○	○
**PATIENT-CENTEREDNESS?** (*Definition*: Providing care that is respectful of and responsive to individual patient preferences, needs, and values and ensuring that patient values guide all clinical decisions.)	○	○	○	○	○
**SAFETY?** (Definition: Avoiding injuries to patients from the care that is intended to help them)	○	○	○	○	○
**TIMELINESS?** (*Definition*: Reducing waits and sometimes harmful delays for both those who receive and those who give care.)	○	○	○	○	○

Maintenance

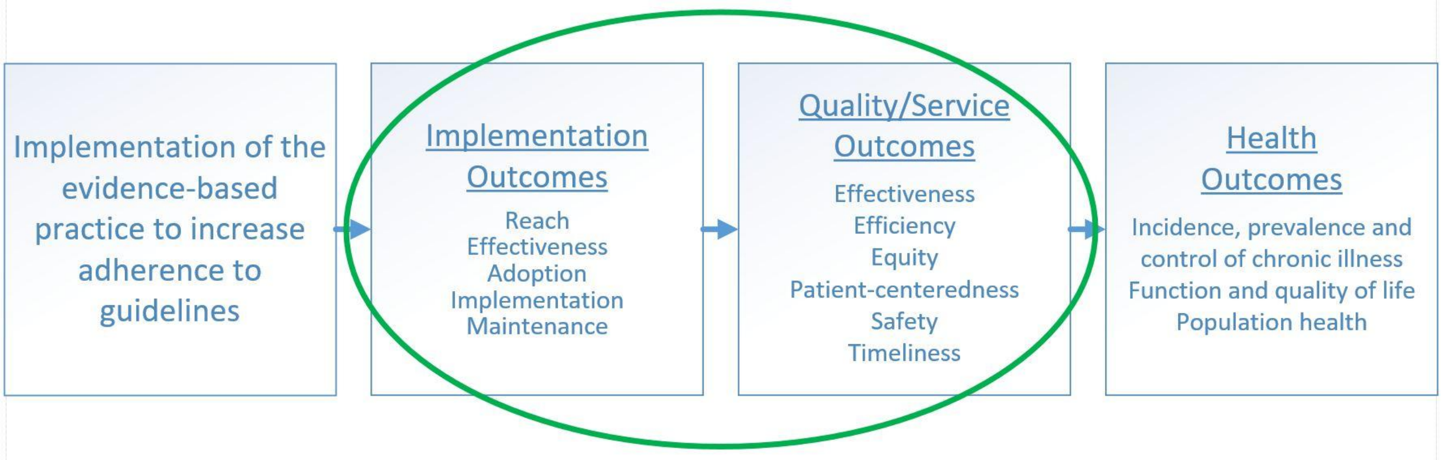

Maintenance

The last set of questions are about how the implementation outcome, MAINTENANCE of the guideline adherence program, may be related to each quality/service outcome. **MAINTENANCE is defined as the proportion and representativeness of settings and staff that that continue to deliver the evidence-based program aimed at increasing adherence to recommended guidelines over time.**

In less technical language, **WHICH** settings and staff continue the program over time?

Think about this evidence-based program being delivered in your setting. As the program is delivered, **if MAINTENANCE increases, what would you generally expect to observe about each of the quality/service outcomes?** (be sure to use the entire scale):

	Decreases A Lot	Decreases A Little	No Change	Increases A Little	Increases A Lot
**EFFECTIVENESS?** (*Definition*: Providing care that is based on systematically acquired evidence demonstrating that a program or intervention produces better outcomes than alternatives—including the alternative of doing nothing.)	○	○	○	○	○
**EFFICIENCY?** (*Definition*: Avoiding waste, including waste of equipment, supplies, ideas, and energy.)	○	○	○	○	○
**EQUITY?** (*Definition*: Providing care that does not vary in quality because of personal characteristics such as gender, ethnicity, geographic location, and socioeconomic status.)	○	○	○	○	○

	Decreases A Lot	Decreases A Little	No Change	Increases A Little	Increases A Lot
**PATIENT-CENTEREDNESS?** *(Definition*: Providing care that is respectful of and responsive to individual patient preferences, needs, and values and ensuring that patient values guide all clinical decisions.)	○	○	○	○	○
**SAFETY?** (Definition: Avoiding injuries to patients from the care that is intended to help them)	○	○	○	○	○
**TIMELINESS?** (*Definition*: Reducing waits and sometimes harmful delays for both those who receive and those who give care.)	○	○	○	○	○
