# Supplementary material for: RE-AIM implementation outcomes and service outcomes: what’s the connection? results of a cross-sectional survey
Source: BMC Health Serv Res. 2023 Dec 15;23:1417. doi: 10.1186/s12913-023-10422-w (PMC10722784; doi:10.1186/s12913-023-10422-w)
Supplement: Supplementary file 2 — Supplementary Material 2: Appendix Table A, P-values > .001 for pairwise comparisons of mean ratings of perceived relationships of RE-AIM outcomes with service outcomes (N = 259) [file 12913_2023_10422_MOESM2_ESM.docx]

**Appendix Table A**

*P-values > .001 for Pairwise Comparisons of Mean Ratings of Perceived Relationships of RE-AIM Outcomes with Service Outcomes (N = 259)*

| Service Outcome and RE-AIM Outcome Pairing | *p*-value in  Pairwise Comparisons |
| --- | --- |
| Effectiveness |  |
| Reach vs. Adaptation | .13 |
| Reach vs. Maintenance | .04 |
| Adoption vs. Maintenance | .99 |
| Equity |  |
| Reach vs. Adoption | .48 |
| Imp/Adaptation vs. Maintenance | .44 |
| Fidelity vs Adaptation | .99 |
| Fidelity vs Maintenance | .99 |
| Efficiency |  |
| Reach vs. Adoption | .02 |
| Reach vs Fidelity | .01 |
| Reach vs Adaptation | .99 |
| Adoption vs. Fidelity | .99 |
| Adoption vs. Adaptation | .99 |
| Adoption vs. Maintenance | .99 |
| Fidelity vs. Adaptation | .54 |
| Fidelity vs. Maintenance | .99 |
| Adaptation vs. Maintenance | .04 |
| Patient-Centeredness |  |
| Reach vs. Adoption | .02 |
| Reach vs. Fidelity | .99 |
| Reach vs. Maintenance | .99 |
| Adoption vs. Fidelity | .08 |
| Adoption vs. Adaptation | .13 |
| Adoption vs. Maintenance | .05 |
| Fidelity vs. Maintenance | .99 |
| Safety |  |
| Reach vs. Adoption | .03 |
| Reach vs. Adaptation | .99 |
| Reach vs. Maintenance | .004* |
| Adoption vs. Adaptation | .01 |
| Adoption vs. Maintenance | .99 |
| Timeliness |  |
| Reach vs. Fidelity | .20 |
| Reach vs. Adaptation | .99 |
| Reach vs. Maintenance | .002* |
| Adoption vs. Fidelity | .32 |
| Adoption vs. Adaptation | .004* |
| Adoption vs. Maintenance | .99 |
| Fidelity vs. Adaptation | .99 |
| Fidelity vs. Maintenance | .99 |
| Adaptation vs. Maintenance | .02 |

*Notes.* For each pairing of RE-AIM outcomes, the *p*-value is provided for the difference in ratings of their relationships with each service outcome. Only *p*-values ≥ .001 are included; all other differences in mean ratings of RE-AIM outcomes’ relationships with service outcomes were statistically significant with *p-*values < .001. Fidelity = Implementation/Fidelity; Adaptation = Implementation/Adaptation.

*Statistically significant with Bonferroni correction (*p* < .01).
