# Supplementary material for: RE-AIM implementation outcomes and service outcomes: what’s the connection? results of a cross-sectional survey
Source: BMC Health Serv Res. 2023 Dec 15;23:1417. doi: 10.1186/s12913-023-10422-w (PMC10722784; doi:10.1186/s12913-023-10422-w)
Supplement: Supplementary file 3 — Supplementary Material 3: Appendix Table B, ratings of perceived relationships of RE-AIM outcomes with service outcomes by respondent subgroups [file 12913_2023_10422_MOESM3_ESM.docx]

**Appendix Table B**

*Ratings of Perceived Relationships of RE-AIM Outcomes with Service Outcomes by Respondent Subgroups*

|  |  | | Service Outcomes | | | | | | | |
| --- | --- | --- | --- | --- | --- | --- | --- | --- | --- | --- |
| RE-AIM Outcomes^a^ and Respondent Groups | | N | | Effectiveness  M(SD) | Efficiency  M(SD) | Equity  M(SD) | Patient-Centeredness  M(SD) | Safety  M(SD) | Timeliness  M(SD) |  |
| Reach | |  | |  |  |  |  |  |  |  |
| High IS experience | | 68 | | 0.71 (1.11) | 0.65 (0.96) | 1.31 (0.74) | 0.65 (1.05) | 0.34 (0.89) | 0.29 (1.04) |  |
| Not High IS experience | | 190 | | 0.95 (1.01) | 0.66 (1.01) | 1.29 (0.83) | 0.54 (0.96) | 0.44 (0.95) | 0.51 (1.07) |  |
| High HSR experience | | 89 | | 0.94 (1.09) | 0.65 (0.99) | 1.30 (0.76) | 0.74 (0.97) | 0.49 (0.98) | 0.62 (1.05) |  |
| Not High HSR experience | | 168 | | 0.86 (1.01) | 0.66 (1.01) | 1.29 (0.84) | 0.60 (0.99) | 0.36 (0.90) | 0.37 (1.06) |  |
| Use RE-AIM frequently | | 75 | | 0.61 (1.16) | 0.55 (1.02) | 1.28 (0.81) | 0.53 (1.02) | 0.28 (0.92) | 0.43 (1.07) |  |
| Do not use RE-AIM frequently | | 183 | | 1.00 (0.97) | 0.70 (0.99) | 1.31 (0.81) | 0.70 (0.97) | 0.46 (0.94) | 0.46 (1.06) |  |
| Clinician | | 32 | | 1.28 (0.96) | 0.90 (1.04) | 1.13 (1.12) | 0.52 (1.09) | 0.52 (1.21) | 0.53 (1.11) |  |
| Non-Clinician | | 226 | | 0.83 (1.04) | 0.62 (0.99) | 1.32 (0.76) | 0.67 (0.97) | 0.40 (0.89) | 0.44 (1.06) |  |
| Adoption | |  | |  |  |  |  |  |  |  |
| High IS experience | | 68 | | 0.94 (0.96) | 0.62 (0.93) | 1.13 (0.71) | 0.74 (0.68) | 0.47 (0.78) | 0.72 (0.86) |  |
| Not High IS experience | | 190 | | 1.08 (1.00) | 0.97 (0.92) | 1.21 (0.80) | 0.89 (0.89) | 0.63 (0.96) | 0.82 (0.99) |  |
| High HSR experience | | 89 | | 1.30 (0.99) | 0.84 (0.98) | 1.20 (0.76) | 0.87 (0.83) | 0.66 (1.02) | 0.90 (0.93) |  |
| Not High HSR experience | | 168 | | 1.02 (0.98) | 0.90 (0.92) | 1.18 (0.79) | 0.85 (0.85) | 0.55 (0.86) | 0.73 (0.97) |  |
| Use RE-AIM frequently | | 75 | | 1.00(1.11) | 0.83 (0.96) | 1.13 (0.70) | 0.76 (0.68) | 0.52 (0.89) | 0.87 (0.86) |  |
| Do not use RE-AIM frequently | | 183 | | 1.16 (0.93) | 0.90 (0.92) | 1.21 (0.80) | 0.89 (0.90) | 0.62 (0.93) | 0.76 (0.99) |  |
| Clinician | | 32 | | 1.41 (0.84) | 1.00 (0.76) | 1.06 (0.89) | 0.81 (1.01) | 0.84 (1.00) | 0.94 (1.01) |  |
| Non-Clinician | | 226 | | 1.08 (1.00) | 0.86 (0.96) | 1.21 (0.76) | 0.85 (0.81) | 0.56 (0.90) | 0.77 (0.95) |  |
| Implementation/Fidelity | |  | |  |  |  |  |  |  |  |
| High IS experience | | 68 | | 1.34 (0.80) | 0.84 (0.96) | 0.68 (0.95) | 0.49 (0.87) | 0.85 (0.81) | 0.44 (0.94) |  |
| Not High IS experience | | 190 | | 1.58 (0.63) | 0.96 (0.99) | 0.89 (0.93) | 0.76 (0.97) | 0.92 (0.84) | 0.71 (0.96) |  |
| High HSR experience | | 89 | | 1.57 (0.64) | 0.93 (0.90) | 0.93 (0.92) | 0.69 (0.89) | 0.89 (0.87) | 0.74 (0.94) |  |
| Not High HSR experience | | 168 | | 1.49 (0.71) | 0.92 (1.03) | 0.79 (0.95) | 0.68 (0.99) | 0.90 (0.82) | 0.58 (0.97) |  |
| Use RE-AIM frequently | | 75 | | 1.39 (0.82) | 0.76 (1.06) | 0.71 (0.88) | 0.55 (0.92) | 0.81 (0.80) | 0.51 (0.96) |  |
| Do not use RE-AIM frequently | | 183 | | 1.57 (0.62) | 1.00 (0.95) | 0.89 (0.96) | 0.74 (0.96) | 0.93 (0.85) | 0.69 (0.95) |  |
| Clinician | | 32 | | 1.47 (0.84) | 1.16 (0.93) | 1.00 (0.86) | 0.58 (0.96) | 1.16 (0.78) | 0.87 (0.98) |  |
| Non-Clinician | | 226 | | 1.53 (0.66) | 0.90 (0.99) | 0.81 (0.95) | 0.70 (0.95) | 0.86 (0.84) | 0.60 (0.95) |  |
| Implementation/Adaptation | |  | |  |  |  |  |  |  |  |
| High IS experience | | 68 | | 0.60 (1.01) | 0.62 (0.90) | 0.84 (0.87) | 0.93 (0.97) | 0.32 (0.82) | 0.41 (0.78) |  |
| Not High IS experience | | 190 | | 0.68 (1.19) | 0.79 (0.98) | 0.76 (0.95) | 1.07 (0.86) | 0.37 (0.97) | 0.57 (0.89) |  |
| High HSR experience | | 89 | | 0.64 (1.10) | 0.71 (0.92) | 0.74 (0.90) | 1.08 (0.86) | 0.30 (0.95) | 0.62 (0.83) |  |
| Not High HSR experience | | 168 | | 0.66 (1.17) | 0.77 (0.99) | 0.80 (0.95) | 1.01 (0.91) | 0.38 (0.92) | 0.48 (0.88) |  |
| Use RE-AIM frequently | | 75 | | 0.72 (1.13) | 0.80 (0.94) | 0.95 (0.84) | 1.09 (0.86) | 0.36 (0.80) | 0.63 (0.79) |  |
| Do not use RE-AIM frequently | | 183 | | 0.63 (1.15) | 0.73 (0.97) | 0.72 (0.96) | 1.01 (0.90) | 0.36 (0.98) | 0.49 (0.89) |  |
| Clinician | | 32 | | 0.75 (1.11) | 0.94 (1.06) | 0.45 (1.15) | 0.87 (0.96) | 0.68 (1.11) | 0.75 (0.92) |  |
| Non-Clinician | | 226 | | 0.65 (1.15) | 0.72 (0.95) | 0.83 (0.89) | 1.06 (0.88) | 0.31 (0.89) | 0.50 (0.85) |  |
| Implementation/Cost | |  | |  |  |  |  |  |  |  |
| High IS experience | | 68 | | 0.06 (0.84) | -0.57 (0.95) | -0.47 (0.99) | -0.19 (0.83) | -0.03 (0.65) | -0.29 (0.90) |  |
| Not High IS experience | | 190 | | 0.19 (0.99) | -0.30 (1.14) | -0.34 (1.12) | -0.05 (0.99) | 0.11 (-0.85) | -0.13 (1.08) |  |
| High HSR experience | | 89 | | 0.16 (1.06) | -0.42 (1.04) | -0.37 (1.05) | -0.15 (0.95) | 0.03 (0.83) | -0.24 (1.10) |  |
| Not High HSR experience | | 168 | | 0.16 (0.90) | -0.34 (1.13) | -0.36 (1.10) | -0.05 (0.95) | 0.10 (0.78) | -0.12 (0.99) |  |
| Use RE-AIM frequently | | 75 | | 0.25 (0.95) | -0.40 (1.04) | -0.31 (1.03) | -0.08 (0.91) | 0.08 (0.78) | -0.23 (1.05) |  |
| Do not use RE-AIM frequently | | 183 | | 0.12 (0.96) | -0.36 (1.13) | -0.40 (1.11) | -0.09 (0.97) | 0.07 (0.81) | -0.15 (1.03) |  |
| Clinician | | 32 | | -0.09 (1.03) | -0.45 (1.29) | -0.58 (1.12) | -0.26 (0.97) | -0.06 (0.93) | -0.28 (1.05) |  |
| Non-Clinician | | 226 | | 0.19 (0.95) | -0.36 (1.08) | -0.34 (1.08) | -0.07 (0.95) | 0.09 (0.78) | -0.15 (1.03) |  |
| Maintenance | |  | |  |  |  |  |  |  |  |
| High IS experience | | 68 | | 0.94 (0.98) | 0.82 (0.81) | 0.81 (0.85) | 0.57 (0.89) | 0.59 (0.82) | 0.60 (0.76) |  |
| Not High IS experience | | 190 | | 1.17 (0.95) | 1.05 (0.91) | 0.99 (0.85) | 0.73 (0.87) | 0.67 (0.90) | 0.80 (0.90) |  |
| High HSR experience | | 89 | | 1.13 (0.91) | 0.92 (0.87) | 0.97 (0.83) | 0.63 (0.84) | 0.70 (0.90) | 0.72 (0.83) |  |
| Not High HSR experience | | 168 | | 1.10 (0.99) | 1.03 (0.90) | 0.92 (0.86) | 0.71 (0.90) | 0.62 (0.86) | 0.77 (0.89) |  |
| Use RE-AIM frequently | | 75 | | 1.01 (0.99) | 0.87 (0.84) | 0.91 (0.86) | 0.59 (0.81) | 0.59 (0.86) | 0.72 (0.74) |  |
| Do not use RE-AIM frequently | | 183 | | 1.15 (0.95) | 1.04 (0.90) | 0.96 (0.85) | 0.73 (0.90) | 0.68 (0.88) | 0.76 (0.91) |  |
| Clinician | | 32 | | 1.16 (1.14) | 0.84 (1.16) | 0.87 (1.15) | 0.58 (1.02) | 0.77 (1.06) | 1.00 (0.92) |  |
| Non-Clinician | | 226 | | 1.10 (0.94) | 1.01 (0.85) | 0.95 (0.81) | 0.70 (0.86) | 0.63 (0.85) | 0.71 (0.86) |  |

*Note.* For each pairing, respondents were asked to select what they would generally expect to be observed in the service outcome if the RE-AIM outcome were to increase (e.g., “As the program is delivered, if REACH increases, what would you generally expect to observe about each service outcome: effectiveness, efficiency, equity, patient-centeredness, safety, timeliness”). Response options were assigned the following values: -2 = Decreases a lot; -1 = Decreases a little; 0 = No change; 1 = Increases a little; 2 = Increases a lot. Scores above 0 indicate a perceived positive relationship between the RE-AIM outcome and the service outcome; scores below 0 indicate a perceived negative relationship between the RE-AIM outcome and the service outcome; and scores near 0 indicate no perceived relationship between the RE-AIM outcome and the service outcome. Potential differences in ratings across four sets of groups were tested: respondents reporting high levels of implementation science experience versus all others; respondents reporting frequently using RE-AIM versus all others; respondents reporting high levels of health services research experience versus all others; and clinicians versus all others. No significant differences (*p* < .05) in mean ratings of relationships were found in these subgroup comparisons.

IS = implementation science. HSR = health services research.

^a^ Effectiveness is a RE-AIM outcome and a service outcome. The survey included Effectiveness as both. In analyses, Effectiveness is used only as a service outcome to decrease redundancy.
